# Battling Neurodegenerative Diseases with Adeno-Associated Virus-Based Approaches

**DOI:** 10.3390/v12040460

**Published:** 2020-04-18

**Authors:** Olja Mijanović, Ana Branković, Anton V. Borovjagin, Denis V. Butnaru, Evgeny A. Bezrukov, Roman B. Sukhanov, Anastasia Shpichka, Peter Timashev, Ilya Ulasov

**Affiliations:** 1 Group of Experimental Biotherapy and Diagnostics, Institute for Regenerative Medicine, Sechenov First Moscow State Medical University, Moscow 119991, Russia; olja.mijanovic@gmail.com; 2 Department of Forensics, University of Criminal Investigation and Police Studies, Belgrade 11000, Serbia; sonny_brankovic@yahoo.com; 3 Department of Biomedical Engineering, University of Alabama at Birmingham, Birmingham, AL 35294, USA; aborovagin@gmail.com; 4 Institute for Regenerative Medicine, Sechenov First Moscow State Medical University, Moscow 119991, Russia; butnaru_dv@mail.ru (D.V.B.); ana-shpichka@yandex.ru (A.S.); timashev.peter@gmail.com (P.T.); 5 Institute for Uronephrology and Reproductive Health, Sechenov First Moscow State Medical University, Moscow 119991, Russia; eabezrukov@rambler.ru (E.A.B.); rb_suhanov@mail.ru (R.B.S.); 6 Institute of Photonic Technologies, Research Center “Crystallography and Photonics”, Russian Academy of Sciences, Troitsk, Moscow 142190, Russia; 7 Department of Polymers and Composites, N.N. Semenov Institute of Chemical Physics, Moscow 119991, Russia; 8 Chemistry Department, Lomonosov Moscow State University, Moscow 119991, Russia

**Keywords:** AAV, neuro-degenerative disease, gene therapy

## Abstract

Neurodegenerative diseases (NDDs) are most commonly found in adults and remain essentially incurable. Gene therapy using AAV vectors is a rapidly-growing field of experimental medicine that holds promise for the treatment of NDDs. To date, effective delivery of a therapeutic gene into target cells via AAV has been a major obstacle in the field. Ideally, transgenes should be delivered into the target cells specifically and efficiently, while promiscuous or off-target gene delivery should be minimized to avoid toxicity. In the pursuit of an ideal vehicle for NDD gene therapy, a broad variety of vector systems have been explored. Here we specifically outline the advantages of adeno-associated virus (AAV)-based vector systems for NDD therapy application. In contrast to many reviews on NDDs that can be found in the literature, this review is rather focused on AAV vector selection and their testing in experimental and preclinical NDD models. Preclinical and in vitro data reveal the strong potential of AAV for NDD-related diagnostics and therapeutic strategies.

## 1. Background

Neurodegenerative diseases (NDDs) are the most common primary pathologies of the central nervous system (CNS) in adults. They affect the majority of the elderly population and arise either as a consequence of an accumulation of previously acquired genetic modification in the human genome or de novo—i.e., without preceding neurodegenerative pathologies—as a result of an accumulation of neurotoxic proteins in the cytoplasm of neurons (de novo disease). The lack of effective therapies to control NDD made the progression of the disease inevitable [1]. Systemic effects from NDD development are usually associated with infiltration of the brain with peripheral immune cells [2], which contributes to neuroinflammation [3] and neurodegeneration [4]. The cause of systemic and molecular abnormalities developing throughout the NDD progression remains to be investigated as any developments in the NDD therapy field require a detailed understanding of the molecular mechanism of neurodegeneration process as well as how to intervene with the disease process.

A significant determinant of NDD progression is an accumulation of toxic proteins on a subcellular level [5,6]. Intracellular accumulation of specific abnormally-aggregated proteins in the form of inclusion bodies is associated with different pathologies of most common NDDs: while Alzheimer’s destroys memory, Parkinson’s and Huntington’s diseases affect movement. Previous research has suggested that all three diseases are caused by the death of neurons and other cells in the brain, which is associated with abnormally-folding and aggregating amyloid proteins, different in each disease. For instance, a misfolded amyloid-β and mutated tau, α-synuclein, huntingtin, and ALS-linked trans-activation response (TAR) DNA-binding protein 43 (*TARDBP*) along with FUS are implicated in molecular mechanisms of Alzheimer’s, Parkinson’s, Huntington’s, and amyotrophic lateral sclerosis (ALS), respectively [7]. Those all form insoluble clumps inside brain cells that ultimately rupture the endocytic vesicles. It was suggested that the latter mechanism is conserved and, therefore, common treatment strategies should be possible that would involve boosting a brain cell’s ability to degrade protein clumps and damaged vesicles [8]. This observation offers a possibility for therapeutic intervention, as it suggests the involvement of only a few signaling pathways that recently emerged as potential targets for neuronal dysregulation. Among them is autophagy signaling, whose dysfunction permits the accumulation of misfolded proteins in various structural parts of the neural cells [9,10]. All the dysregulation results in excessive inhibition of adenosine triphosphate (ATP)-dependent chaperones and proteolytic machinery [11] that represses neurogenesis [12], stimulates damage via oxidative stress [13], and promotes neuronal cell death. Recent studies suggest that inactivated autophagy-related proteins in damaged neurons [14] represent a suitable target for experimental NDD therapy [15]. Although there is extensive literature on the accumulation of toxic proteins inside neuronal cells as a result of autophagy repression and its pharmacological correction [16], relatively few studies have addressed the possibility of targeting neuronal cells with aberrant signaling [17,18,19,20,21] by using alternate approaches, such as gene therapy. Below, we summarized the benefits of gene therapy application for NDD treatment.

Neurodegenerative diseases are typically caused by numerous genetic abnormalities, whose effect is enhanced by environmental factors [22] and epigenetic events [23,24]. Basic research has identified numerous pathways that contribute to NDD pathogenesis. Based on this rapidly growing knowledge, multiple drugs for NDD therapy have been approved to-date [25,26,27,28,29]. Interestingly, Chinese herbal medicine may also represent a promising alternative for NDD treatment [30]. In the past three decades, the scientific community has made significant efforts in utilizing newly developed molecular technologies to selectively deliver a therapeutic payload into target cells via a nonconventional method, known as “gene therapy” [31,32].

Viral vector-based gene therapy offers a new therapeutic opportunity against an irreversible lethal impact of many inheritable or genetic diseases. For instance, in the case of Spinal Muscular Atrophy type 1 (SMA1), a single dose of intravenously delivered AAV9 vector carrying a transgene encoding the missing SMN protein resulted in longer survival of patients [33]. Another example is the study by Massaro et al., who used a mouse model of an untreatable acute form of infantile neuronopathic Gaucher disease to inject an adeno-associated virus (AAV)-based vector encoding neuronal glucocerebrosidase that abolished neurodegeneration and ameliorated neuroinflammation [34]. In a later study, gene therapy with an AAV vector improved motor skills and increased lifespan of animals in a mouse model of inherited lysosomal storage disorders, known as the neuronal ceroid-lipofuscinoses (NCLs) or more commonly referred to as Batten disease, a form of NCLs with a deficiency in the membrane-bound protein CLN6 (CLN6 disease) [35]. Although it is too early for evaluating the clinical efficacy of the developed AAV gene therapy vectors in human patients, some AAV-based gene therapies demonstrate a remarkable therapeutic effect in preclinical studies. For example, an AAV-based gene therapy aimed at correcting a rare metabolic aromatic L-amino acid decarboxylase deficiency (AADC) was granted a biologics license application (BLA)-ready status by the FDA in 2018 [36].

## 2. Viral Vector-Based Gene Therapy: Efficiency and Selectivity of Delivery

### 2.1. Choosing an Ideal Vector

Gene therapy for NDDs represents a rapidly developing novel therapeutic approach with a potential capability of restoring normal neuronal functions [37] by intracellular delivery of exogenous genetic material, carrying a disease correcting/therapeutic information in the form of recombinant deoxyribonucleic acid (DNA) or ribonucleic acid (RNA) molecules engineered into a delivery vehicle, known as vector. Once inside a recipient (host) cell, the transferred genetic material utilizes cellular machinery to biosynthesize (translate the encoded information into) therapeutic proteins aimed at replacing their defective counterparts, produced in neurons by mutated endogenous gene, inactivating the mutated gene’s product or inserting a foreign transgene with therapeutic properties to battle the disease. Despite the variability in the existing delivery vectors, routes of delivery, and delivery techniques, the intracellular mechanism of the delivered gene’s expression remains essentially the same. The transduced or transfected host cells then begin synthesizing protein(s) aimed at repairing a congenital disorder or treat an acquired disease [38]. Depending on the nature of target cells, gene delivery can be classified as either somatic or germline. While the goal of somatic gene delivery is to directly express the transgene in target cells and correct the disease at the individual cell level [39], germline gene transfer is aimed at integrating a therapeutic or correction gene (fully functional wild type copy) into the germline cells, thereby creating inheritable changes, affecting every cell of the next generation. Although more advanced techniques of gene delivery are constantly being developed, the most common approach to correct NDDs involves a direct transfection or transduction (when using virus-based delivery vectors) of isolated patient-derived cells or modification of target cells in situ—i.e., directly in patient’s tissue. The former strategy of gene delivery, known as ex vivo gene therapy, involves isolation and in vitro culturing of patient-derived cells for exogenous genetic modification or gene correction and their subsequent injection back into the patient [40]. In contrast, in vivo gene therapy involves the delivery of genetic modifications to target cells directly by systemic [41]—i.e., intraperitoneal (ip) [42,43] or intravenous (iv) [44,45,46] vector administration. However, large doses of systemically-delivered AAV vectors elicit immune responses affecting transgene expression, treatment longevity and even patient safety. To circumvent those drawbacks, alternate routes of AAV administration to the central nervous system (CNS) have been explored that allowed administration of lower doses of the gene therapy vector directly by into the brain parenchyma or by injection into the cerebrospinal fluid via the intracerebroventricular or intrathecal (cisternal or lumbar) routes. This strategy results in higher transgene expression along with the decreased immune responses. However, the remaining critical concern of the direct CNS-delivery is AAV-associated neuroinflammation, manifested in preclinical studies by the dorsal root ganglion (DRG) and spinal cord pathology with mononuclear cell infiltration [47].

Since the precision of neural cell targeting as well as the expression magnitude of a therapeutic payload are critical characteristics for the efficient repairing of genetic or metabolic abnormalities, the use of viral vector-based gene therapy, well-suited for modulation of those characteristics, offers a significant advantage over the utilization of non-viral vectors.

Due to an essential condition for an effective gene therapy intervention is an efficient and specific delivery of a therapeutic transgene to target cells, an ideal vector should be inherently suitable for or possibly tailored (genetically engineered) to specific target cells of interest. In addition, it should not trigger a robust immune response in the host and be capable of providing a sustained transgene expression for the duration of disease treatment [48]. By these criteria, some virus-based vectors became preferred gene delivery vehicles in contemporary gene therapy applications. Since the natural mechanism of viral cell entry involves attachment to target cell’s surface receptor(s), most virus-based gene therapy vectors can be engineered by retargeting the viral particle (capsid) surface to primary receptors abundant on or even specific to target cells by using genetic and other molecular technologies developed in the past two decades. Viral gene therapy vectors specifically designed for NDD applications should be able to efficiently cross the blood–brain barrier (BBB), if delivered by iv infusion [44,49], and support an efficient transgene expression, regardless of aberrant cell signaling caused by the accumulation of toxic proteins or restoration of an autophagic state in neuronal cells [50]. For instance, vectors based on genomes of adeno-associated virus (AAV) [44,49,51,52,53,54,55,56], lentivirus [57], type 1 herpes simplex virus [58], and Semliki Forest virus (SFV) [59] have been broadly used for neuron targeting. Extensive use of the above viruses in neuroscience is dictated by their natural ability to efficiently and selectively transduce and provide high transgene expression in neurons. Moreover, those viral vectors minimally perturb stress pathways in the infected neural cells [60,61,62], which makes them attractive tools specifically for the NDD gene therapy applications. When the efficiency and the safety of AAV and lentiviral vectors were compared, AAV demonstrated a significantly higher transgene expression levels and minimal interaction with the innate immune effectors. In contrast, lentiviral transduction resulted in modest transgene expression efficiency and provoked a rapid self-limiting proinflammatory response [63].

In contrast to the majority of viral vectors utilized for gene therapy interventions, AAV-based vectors exhibit the highest potential and success rate in the treatment of several monogenic diseases, as demonstrated by clinical trials [64,65].

### 2.2. Advantages of AAV-Based Vectors

What makes AAV an exceptional candidate vector for NDD gene therapy applications is its low pathogenicity in humans: none of the AAV subtypes appear to cause a life-threatening systemic response in humans. This is an important feature since patient safety is the most critical condition for the successful development of gene therapy interventions of NDD and other human diseases. Natural tropism of most AAV serotypes, particularly that of AAV2, towards various human tissue types that include the retina and neural tissues [66], liver [67,68], vascular tissue [69,70], lung [71], and skeletal muscles [72] is the other important criterion making AAV a preferred gene therapy vector for NDD [73,74]. The broad tropism of AAV is owed to the diversity of its natural cell-binding targets, such as primary receptors heparan sulfate proteoglycan (HSPG) [75] and/or *N*-glycans with terminal galactose [48], and several known co-receptors, such as fibroblast growth factor (FGF) receptor 1 [76], platelet-derived growth factor (PDGF) receptor [77], hepatocyte growth factor receptor [78], α_V_β_1_ and α_V_β_5_ integrins [79,80], O-linked sialic acid [81] and laminin receptor (LRP/LR), previously found in liver tissue [82], and recently also discovered in neurons [83]. In fact, cell surface receptors have been definitively identified only for some AAV serotypes: HSPG for AAV-3, O-linked sialic acid for AAV4, PDGF for AAV5, and LRP/LR for AAV serotypes 2, 3, 8, and 9 [49]. To date, there have been 11 identified AAV serotypes (AAV1-AAV11). Due to the fact that the transduction of human neural cells with AAV2 serotype is limited, and humans are seropositive with regard to AAV2, the other AAV serotypes have been explored as alternative gene therapy vectors. This allowed achieving more efficient transduction of selective subsets of brain cells in animal NDD models [84].

The versatility of AAV tropism can be attributed to variability in the amino acid (aa) sequence of the AAV structural (capsid) proteins VP1, VP2, and VP3, whose mixture (in the ratio of 1:1:10, respectively) makes icosahedral symmetry capsid of 60 monomers that are involved in cell surface attachment of the viral particles. The variability in the amino-acid (aa) sequence is confined to the 9 surface-exposed (VR-I to VR-IX) domains of the AAV structural/capsid proteins. AAV tropism alteration for tailoring of the vectors to specific gene therapy applications can be achieved by virus pseudotyping—i.e., creating artificial hybrid AAV particles by combining genome from one strain/serotype with capsid proteins from a different strain/serotype [85,86]. AAV capsid engineering is a two-decade-old approach that embodies two distinct strategies: directed evolution and rational design. The former involves shuffling of capsid genes from available serotypes [87], random peptide insertion into known sites of AAV capsid [88,89] or phage display [90] and ultimately involves a selection process that requires multiple generations of screening to identify functional capsids. Alternatively, a rational design strategy utilizes a structural knowledge to refine capsid structure and/or replace native cell-binding motifs with foreign motifs of desired affinity [90,91,92,93]). Recently, a more advanced method of AAV targeting, known as barcoded rational AAV vector evolution (BRAVE), was proposed that encompasses all of the benefits of the rational design approach while maintaining the broad screening diversity permitted by directed evolution, but with only a single generation screening. This new strategy is based on the viral library production approach, where each viral particle displays a protein-derived peptide on the surface, which is linked to a unique barcode in the packaged genome [94].

Naturally, AAV does not produce replication factors and, therefore, is unable to replicate (propagate) without a helper virus (adenovirus, herpes simplex virus (HSV) or vaccinia virus), which provides activating proteins to AAV to enable its replication. Furthermore, in the presence of some helper viruses, such as adenovirus, AAV can even exhibit a lysogenic behavior, which can rarely occur without a helper. Replication deficiency of AAV adds to the biological safety of AAV as yet another benefit for its application as a gene therapy vector.

## 3. AAV in NDD Experimental Therapy

### 3.1. In Vitro vs. In Vivo Models

With regard to NDD gene therapy, an experimental tissue model for the blood-brain barrier (BBB) is of the highest relevance and importance, and the most developed disease models include those for Alzheimer’s, Parkinson’s, and Huntington’s diseases. These and other NDD models are discussed in detail elsewhere [95]. Numerous NDD disease platforms have been created to date by using various approaches [96,97,98,99]. This includes several in vitro disease models developed in the last three years, which were successfully used in a few NDD gene therapy studies. One of the crucial criteria in the efficacy assessment of viral vector-based gene therapy of NDD is the ability of delivery vectors to efficiently cross the BBB, as iv administration remains the primary route of vector delivery. In contrast to in vivo (animal) models, in vitro models allow us to easily understand the mechanism of viral trafficking. In this regard, Merkel et al. showed that primary human brain microvascular endothelial cells, as a model of the human BBB, can be effectively utilized as a tool to characterize trans-endothelial movement and transduction kinetics of various AAV serotypes in vitro and even their effect on BBB integrity. Specifically, by using this in vitro BBB model, the authors found that AAV9 penetrates brain microvascular endothelial cell barriers more effectively than AAV2, although it exhibits relatively lower transduction efficiency [100].

A proper design of NDD in vivo models should allow assessing the robustness of therapies in the settings that mimic clinical endpoints. Although information obtained from studying in vitro models is usually capable of providing a sufficient understanding of disease pathogenesis, it fails to characterize the aspect of disease progression. Therefore, the use of in vivo disease models allows investigators to observe and analyze symptomatic manifestation of NDDs alongside with the therapeutic outcomes of their experimental treatment. This also aids in getting a comprehensive insight into the complex interconnection between etiological and symptomatic changes during the progression of NDDs, such as neuronal ceroid lipofuscinosis (NCL or Batten disease) [35], spinal muscular atrophy [101], or Niemann-Pick disease [102], whose development is typically associated with genetic perturbations in multiple genes. Therefore, in contrast to in vitro models, a mouse model of NDD would allow recapitulating onset and progression of the counterpart human disease of a given degree of severity. For instance, the studies involving AAV9-based vectors [103,104,105,106] aimed at restoring expression of target genes with genetic abnormalities require testing of the experimental strategies in a preclinical setting and under different routes of vector delivery to affected cells. Hudry et al. [49] were able to evaluate the efficacy of AAV-mediated intracellular delivery of reporter transgene in a CNS disorder. It was found that, depending on the viral capsid composition and the route of vector administration, AAV9 is capable of providing a high level of transduction in mixed astrocyte and neuron culture, although vector biodistribution upon intracerebroventricular injection can vary. As far as the route of injection is concerned, a direct injection of experimental therapeutics into cerebrospinal fluid allows their fast and efficient delivery and spread throughout CNS and spinal cord. Therefore, intrathecal injection is nowadays commonly used in various experimental strategies as an effective route of viral agent delivery and achieving therapeutic effects [107,108,109,110].

### 3.2. Selection of Optimal Gene Therapy Strategy

**Figure 1:**
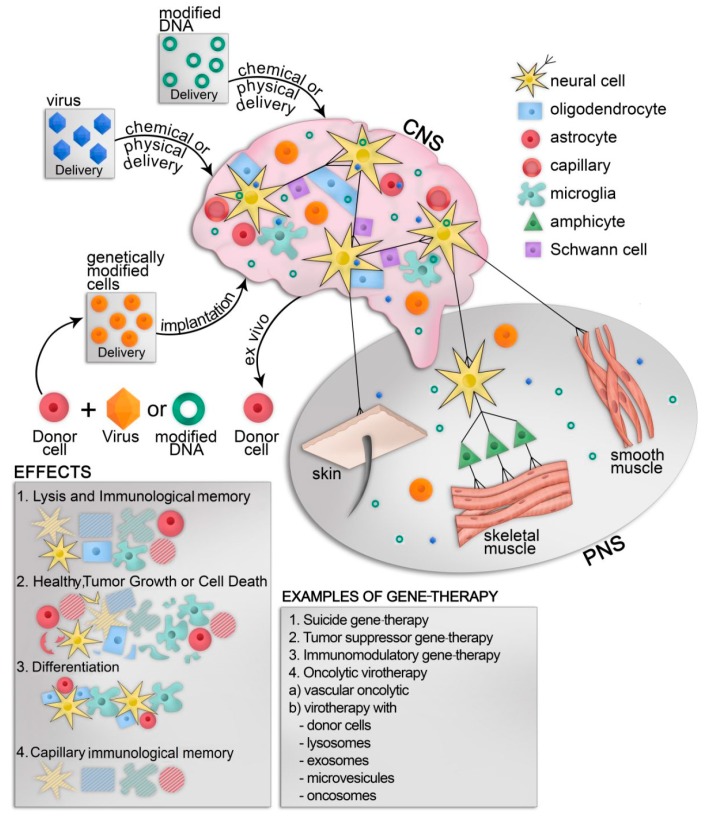
Potential applications of gene targeting for therapy of neurodegenerative diseases.

The choice of a suitable gene therapy strategy for NDD is commonly dictated by the known alterations in signaling pathways associated with a particular NDD. Multiple scientific reports suggest that under normal physiological conditions a microtubule-associated protein tau (MAPT) is sensitive to autophagy-associated ubiquitin-binding protein p62/SQSTM1, which promotes degradation of the misfolded, microtubule-dissociated tau protein. However, in tauopathy, an insoluble form of mutant MAPT becomes resistant to recognition by and binding to p62/SQSTM1 and as a result, fails to degrade inside autophagosomes [6]. This study revealed that the pathology caused by the intracellular formation of neurofibrillary tangles (NFTs) by the hyperphosphorylated form of mutant MAPT, can be suppressed by p62/SQSTM1 overexpression resulting from delivery of AAV 2/9-SQSTM1 expression vector. Earlier, Janda et al. demonstrated that several NDDs are associated with an onset of oxidative stress that inhibits LC3 lipidation and autophagic flux in the affected neurons [111], suggesting multiple reasons for the reduction of basal autophagy and the resulting promotion of NDD. Since the discovery of the relevance of defective autophagy signaling to NDDs, a few studies utilized AAV vectors to overcome the autophagy signaling defects. It was reported that defects in autophagy signaling can be overcome by activation of lysosomal transport via intracellular expression of hydrolase glucocerebrosidase [112] or reduction of tau protein phosphorylation via arginase 1 [113] that eliminates the cargo from vulnerable neurons. In the aggregate, the above-referenced studies strongly suggest that rescuing impairments in the autophagy pathway inside diseased cells is a promising therapeutic strategy for some NDDs. Moreover, aberrant signaling that is attributed to the NDD progression can also be corrected via AAV-based vectors. Recently, it was shown that the expression of triggering receptor expressed on myeloid cells 2 (TREM2) is associated with high risk for AD development. Therefore, AAV-mediated expression of the soluble form of TREM2 (sTREM2), a proteolytic product of TREM2, reduces amyloid plaque load and rescues functional deficits of spatial memory [114]. Overall, the delivery of a therapeutic payload via a modified AAV contributes to the restoration of the disease-affected pathway (Figure 1).

### 3.3. AAV-Based Gene Therapy for Parkinson’s Disease

The movement disorder, known as Parkinson’s Disease (PD), the second most common neurodegenerative disorder, occurs largely due to the loss of dopaminergic neurons of the substantia nigra, resulting in striatal depletion of the neurotransmitter dopamine. AAV-based gene therapy vectors could be effectively used for gene therapy of PD through increasing dopamine levels in target cells [115]. Another strategy proposed for gene therapy of PD is based on targeting of neural parenchyma by AAV-based α-synuclein expression vector (AAV-PHP.B-GBA1) delivered via an iv administration, as opposed to commonly used injection in the mouse forebrain. This allowed vector to permeate and diffuse throughout the neural parenchyma, resulting in targeting of both the central and the peripheral nervous systems in a global pattern and recovering animal behavior by reducing synucleinopathy [44].

Gutekunst et al. demonstrated that the delivery of C3-ADP ribosyltransferase (C3) selectively and irreversibly inhibits activation of RhoA GTPase (a key intracellular regulator of axon regrowth in the mammalian CNS) in neurons or neuronal progenitors and effectively prevents inhibition of axonal regrowth leading to axon regeneration [116]. Furthermore, the use of AAV-mediated C3 delivery allowed the authors to determine a critical C3 concentration promoting neuron outgrowth on chondroitin sulfate substrate. Another example of AAV vector system implementation for gene therapy of PD is the delivery of rat ATP6V0C (a pore-forming stalk c-subunit of the V0 sector of the vacuolar proton ATPase contributing to release of serotonin, acetylcholine, and dopamine by stromal cells) into the substantia nigra of the diseased mice, in which high potassium stimulation increased overflow of the endogenous dopamine (DA) [117,118].

### 3.4. AAV Vectors and Gene Targeting

In addition to gene delivery for therapeutic protein expression/overexpression purposes in neural cells, AAV vectors have also been used for gene targeting (knockdown and knockout) applications, which include shRNA and miRNA expression strategies. It becomes increasingly popular to knockdown the expression of cellular factors or enzymes contributing to NDD pathobiology to restore normal physiological conditions in disease-affected neural cells. For instance, an AAV8-based vector has been utilized to modulate the expression of cellular proteins implemented in the pathology of the disease. Lu et al. [119] demonstrated that an elevated level of SM synthase-1 promotes the lysosomal degradation of BACE1. However, due to the dominant-negative nature of some genetic mutations, direct sequestration of aberrant (mutant) proteins with normal function-inhibiting properties or even genetic knock-out of mutant genes (excision/destruction of mutant genes) or their complete replacement with corrected/wild type counterparts becomes a necessary and the most rational strategy.

Mutations in genes that control normal CNS development and metabolism give rise to several genetic (inheritable) NDDs. For instance, Krabbe disease is caused by a genetic abnormality that is associated with mutations in the galactosylceramidase (GALC) gene. The GALC enzyme is contained inside lysosomes, where it hydrolyzes specific galactolipids, including galactosylceramide and psychosine (galactosylsphingosine). While the breakdown of those lipids is part of the normal myelin turnover, accumulation of psychosine metabolite in the absence of galactosylceramidase is toxic for neurons and needs to be prevented by a quick restoration of the galactosylceramidase function. Gene therapy for Krabbe disease can thus be accomplished through the GALC gene correction/replacement strategy in the mouse model [46] as well as in the tissue of human patients.

Mutations in superoxide dismutase 1 gene (SOD1) are known to be one of the causes for amyotrophic lateral sclerosis (ALS), a fatal neurodegenerative disease caused by progressive loss of upper and lower motor neurons in human CNS. Although responsible for only about 20% of familial (inherited) ALS cases, *SOD1* mutations contribute to the loss of motor neurons during ALS progression via a toxic gain of function mechanism that involves NF-κB-dependent mechanism, resulting in intracellular aggregation of the aberrant protein [120]. A strategy using an AAV9 delivery vector, engineered to express shRNA targeting SOD1 mRNA, was employed by Frakes et al. to reduce (knock-down) SOD1 expression in both astrocytes and motor neurons. The AAV9 vector was able to efficiently penetrate the BBB and increase the survival of the ALS mice [121]. The same vector was used in the AAV9-mediated knockdown of neuritin, which resulted in the reduction of synaptic transmission in the medial prefrontal cortex (mPFC) pyramidal neurons in mice [122]. Selective suppression of mutant huntingtin aggregation and neuronal dysfunction in a rat model of Huntington’s disease (HD) by applying AAV5-miHTT-451, induced functional improvements in the HD pathological process without causing an activation of microglia or astrocytes immune response [123].

Despite the remarkable therapeutic efficacy of RNAi-based gene silencing in target cells, most of the shRNA-mediated NDD gene therapy applications only partially reduce the *SOD1* gene expression, since neither a complete knockout of the target mRNA [124,125,126], nor a sustained gene knockdown can be technically achieved [124,127]. Therefore, the novel gene targeting technologies, based on the recent discovery of transcription activator-like effector nucleases (TALENs) and clustered regularly interspaced short palindromic repeat (CRISPR)-Cas9 nuclease complex (CRISPR-Cas9 nuclease) as components of bacterial natural “immunity” against viruses, completely revolutionized the genome editing [128] field by dramatically improving gene targeting efficiency and specificity, thereby allowing a highly accurate cleavage of any DNA sequence at any given point [129]. The precision of DNA cleavage mediated by CRISP-Cas9 nucleases has been validated in recent studies aimed at correcting genetic mutations on a single nucleotide level [130]. Wen et al. reported that the deletion of the human insulin growth factor-1 (IGF-1) gene, implicated in hyperpolarization of mitochondrial electrical transmembrane potential, resulted in the accumulation of anti-apoptotic protein factors B-cell lymphoma-extra large (BCL-XL) and B-cell lymphoma 2 (BCL-2). Moreover, the deletion of the *IGF1* gene via an intratracheal injection of an AAV-delivered CRISPR-Cas9 expression system restored mitophagy and reduced protective effect on mitochondria, suggesting a new strategy for treating ALS [131]. A strong therapeutic effect was reported by Raikwar et al. [131] and Gaj et al. [132], who used the CRISPR-Cas9 gene targeting (Figure 1) approach to achieve a reduced expression of the glial maturation factor in glial cells that contributed to the formation of Alzheimer’s disease plaques and corrected an autosomal mutation in the *SOD1* gene, responsible for ALS progression, respectively. Most recently, Ekman et al. pointed out that correction of mutant exon 1 of the huntingtin gene (HTT) results in a ~50% decrease of neuronal inclusions and improved motor deficits [133].

Recently, it was suggested that downregulation of some miRNAs may be involved in modulation of apoptotic response and autophagy defects during the NDD progression [134,135]. Miyazaki et al. found that bulbar muscular atrophy (SBMA) is caused by the expansion of the polyglutamine (poly Q) tract in the androgen receptor (AR-poly Q) protein sequence [136]. It was predicted that several miRNAs, such as miR-196a [136], miR-298 or miR-328 [137], might regulate the activity of NDD-related proteins, such as Elav-like family member 2 (CELF2) that enhances the stability of AR mRNA, or beta-amyloid precursor protein-converting enzyme (BACE), respectively. AAV-based delivery and expression of miR-196a promotes decay of the AR mRNA by silencing its stabilizing factor CUGBP, an Elav-like family member 2 (CELF2), and thereby ameliorates the SBMA phenotypes in the mouse model. These studies suggest that AAV vector is a highly efficient tool for the delivery and the expression of recombinant DNA or regulatory, mRNA-silencing miRNAs in monogenic inherited disease applications. Furthermore, in the case of polygenetic NDD diseases, utilization of AAV for targeting a mutant allele to silence the expression of a mutant protein in patients could become a potent therapeutic approach in the future. One of the remaining challenges of such gene silencing strategy that should be addressed in AAV vector design is how to avoid targeting of the wild type or normal (non-mutated) gene copy in the diseased cells.

## 4. Modulation of Interaction Between the CNS and the Environment

Inflammation is an essential hallmark of various neurodegenerative diseases. An increasing number of clinical studies demonstrate that upon activation microglia and astrocytes produce proinflammatory chemokines, such as tumor necrosis factor alpha (TNFα) and interleukin type 1 (IL1), around amyloid plaques. In most of those cases, administration of interleukin type 2 (IL2) and interleukin type 4 (IL4) has been shown to stimulate an anti-inflammatory response. On the other hand, multiple lines of evidence suggest that the delivery of growth factors via viral vectors can provide significant therapeutic effects. For instance, the nerve growth factor (NGF) was expressed in the brain cells of patients with AD by means of AAV2-based vector delivery in a clinical trial conducted by Tuszynski et al. (Trial Registration: NCT00087789 and NCT00017940). It was found that neurons of the degenerating brain retain the ability to respond to the growth factor administration by axonal sprouting, cell hypertrophy, and activation of functional markers for up to 10 years [138]. Other studies used either lentiviral vector- or AAV-based delivery of a brain-derived neurotrophic factor (BDNF), progranulin (PGRN), or cerebral dopamine neurotrophic factor (CDNF) in mouse models of AD or PD, respectively [71,72,73]. Delivery of BDNF in transgenic APP mouse model (mutant mice carrying two amyloid precursor protein (APP) mutations associated with early-onset familial Alzheimer’s disease) resulted in significant amelioration of cell loss by BDNF and marked improvements in the hippocampal-dependent behavior (contextual fear conditioning), compared with control-treated APP mice [72]. Elevation of PGRN expression in nigrostriatal neurons upon AAV-mediated PGRN delivery protected nigrostriatal neurons from MPTP toxicity, reduced inflammation and apoptosis, as well as completely preserved locomotor function in the mouse PD model [71]. Striatal delivery of AAV2.CDNF allowed recovery of 6-OHDA-induced behavior deficits and resulted in a significant restoration of tyrosine hydroxylase immunoreactive (TH-ir) neurons in the substantia nigra as well as functional recovery of dopaminergic neurons.

An impressive therapeutic outcome was observed in yet another innovative cell/gene therapy combinational approach utilizing the human umbilical cord blood cells (hUCBCs) transduced with adenoviral (Ad) vectors encoding human vascular endothelial growth factor (VEGF), glial cell-line derived neurotrophic factor (GDNF) [139] and/or neural cell adhesion molecule 1 (NCAM) genes [74,75,76]. After hUCBCs were transplanted into transgenic amyotrophic lateral sclerosis (model) mice a significant improvement in animals’ behavioral performance (open-field and grip-strength tests), as well as an increased life-span were observed. Expression of NCAM-VEGF or NCAM-GDNF was observed in ALS mice 10 weeks after delivering genetically modified hUCBCs, whereas the cell vehicles were detectable for 5 months following the transplantation [140].

Although some NDDs are caused by dysregulation of neuron’s synaptic function, AVV-based gene therapy could still offer a therapeutic solution. Specifically, an AAV-mediated delivery of the retinoschisin transgene resulted in the restored structure and function of the photoreceptor cell synapse in the mouse disease models. This restoration of operational synapse in the animal model led to a human clinical trial for gene therapy of X-linked retinoschisis [141]. A recent study established the formation of synaptic connections between NeuroD1-converted neurons with already present neurons. These results indicate possibilities of NeuroD1 AAV-based gene therapy for functional brain repair after ischemic injury through in vivo astrocyte-to-neuron conversion [142].

## 5. Conclusions and Future Directions

Outcomes of clinical trials for NDDs can be compromised by limited data from the prior animal (preclinical) studies, by suboptimal expression vector’s cassette design or by the immunogenicity of either delivery vector itself, or its expression content (a therapeutic transgene). The Clinicaltrials.gov database contains information about 6128 clinical trials worldwide that were aimed at treating various neurodegenerative diseases, including 103 trials using gene therapy approaches 29 of which utilized natural mechanisms of viral replication [143]. At this point, two clinical trials (NCT02418598 and NCT00643890) have been terminated by the organizers (both due to financial reasons) and one (NCT03381729) was suspended by the FDA. The latter study involved 27 patients selected out of 51 with spinal muscular atrophy 1 (SMA1) disorder to receive an injection of AAV9-based vector (6 × 10^13^, 1.2 × 10^14^ and 2.4 × 10^14^ vp per patient) designed to express the SMN protein under the control of hybrid CMV/β-actin promoter (ZOLGENSMA). Currently, the future of ZOLGENSMA use via intrathecal route remains unclear, but the available clinical safety information suggests using caution, when infusing ZOLGENSMA intravenously, and inclusion pre-treatment with corticosteroids, checking blood biochemistry and liver enzymes (https://www.zolgensma.com/how-zolgensma-works). Compared to non-viral vectors (e.g., polyplexes [144]), AAV vectors appear to be highly suitable for NDD gene therapy applications and, therefore, became highly popular for NDD gene therapy studies in the recent years. However, the problem with AAV crossing BBB along with the vector’s immunogenicity [47] in human patients remains the main obstacle even for those vectors that are capable of effectively preventing NDD progression.

In this review, we outlined the major basic and clinical research directions in the field of NDD gene therapy using viral vectors, and particularly AAV, for delivery of therapeutic payload aimed at restoration of autophagy and metabolic defects in neurons. Encouragingly, many of the reported studies demonstrate strong and long-lasting therapeutic effects (Table 1). The summarized basic research data appears to be in good agreement with the results of preclinical studies, suggesting strong efficacies of neuronal cell targeting with AAV. In addition, findings from several basic studies suggest existence of a crosstalk between the brain microenvironment and neuronal cell signaling. A combination of AAV-based gene therapy with other therapeutic strategies targeting autophagy signaling components could result in even more favorable outcomes. Such co-therapeutics include traditional and herbal medicines [145]. Therefore, dysfunction in neuronal signaling is closely interconnected with the brain environment, and a combinational intervention using these two disease components could become a promising therapeutic strategy, which may provide a clue for an effective solution for the ultimate NDD cure.

## Figures and Tables

**Table 1:** Worldwide trials using AAV vectors against neurodegenerative disease.

Vectors	Transgene	Disease	Phase	Patients Enrolled	Outcome	Study name	Clinicaltrail.gov	Status
AAV	Beta-nerve growth factor (NGF)	Alzheimer’s disease	1	10	A Phase I, Dose-Escalating Study to Assess the Safety and Tolerability of CERE-110 [Adeno-Associated Virus (AAV)-Based Vector-Mediated Delivery of Beta-Nerve Growth Factor (NGF)] in Subjects with Mild to Moderate Alzheimer’s Disease	CERE-110 in Subjects With Mild to Moderate Alzheimer’s Disease	**NCT00087789** CERE-110 2.0 × 10^10^ vg, CERE-110 1.0 × 10^11^ vg, CERE-110 2.0 × 10^11^ vg	Completed
AAV	Beta-nerve growth factor (NGF)	Alzheimer’s disease	2	49	A Double-Blind, Placebo-Controlled (Sham Surgery), Randomized, Multicenter Study Evaluating CERE-110 Gene Delivery in Subjects with Mild to Moderate Alzheimer’s Disease	Randomized, Controlled Study Evaluating CERE-110 in Subjects With Mild to Moderate Alzheimer’s Disease	**NCT00876863** CERE-110 2.0 × 10^11^ vg	Completed
AAV	Neurotrophic (growth) factor (Neurturin)	Parkinson’s disease	1/2	60 est/57 fact	A Phase 1/2 Trial Assessing the Safety and Efficacy of Bilateral Intraputaminal and Intranigral Administration of CERE-120 (Adeno-Associated Virus Serotype 2 [AAV2]-Neurturin [NTN]) in Subjects with Idiopathic Parkinson’s Disease	Safety and Efficacy of CERE-120 in Subjects With Parkinson’s Disease	**NCT00985517** CERE-120 2.4 × 10^12^ vg	Completed
AAV	Glutamic acid decarboxylase (GAD)	Parkinson’s disease	1	12	Phase I Study of Subthalamic GAD Gene Transfer in Medically Refractory Parkinson’s Disease	Safety Study of Subthalamic Nucleus Gene Therapy for Parkinson’s Disease	**NCT00195143**	Completed
AAV	Glutamic acid decarboxylase (GAD)	Parkinson’s disease	2	44 (est)	Phase 2 Safety and Efficacy Study Evaluating Glutamic Acid Decarboxylase Gene Transfer to Subthalamic Nuclei in Subjects with Advanced Parkinson’s Disease	Study of AAV-GAD Gene Transfer Into the Subthalamic Nucleus for Parkinson’s Disease	**NCT00643890** One-time bilateral administration of rAAV-GAD at 1 × 10^12^ vector genomes in 35 uL	Terminated (Financial reasons)
AAV	Glutamic acid decarboxylase GAD	Parkinson’s disease		40 est/0 fact	N/A	Long Term Follow-Up Study for rAAV-GAD Treated Subjects	**NCT01301573**	Terminated (Financial reasons)
AAV	Aromatic L-amino acid decarboxylase (hAADC-2)	Parkinson’s disease	1	10	A Phase1 Open-Label Safety Study of Intrastriatal Infusion of Adeno-Associated Virus Encoding Human Aromatic L-Amino Acid Decarboxylase (AAV-hAADC-2) in Subjects with Parkinson’s Disease [AAV-hAADC-2-003]	A Study of AAV-hAADC-2 in Subjects With Parkinson’s Disease	**NCT00229736** 9 × 10^10^ vector genomes (vg) of AAV-hAADC-2 in a single dose of 200 µL bilaterally infused over 4 striatal targets 3 × 10^11^ vector genomes (vg) of AAV-hAADC-2 in a single dose of 200 µL bilaterally infused over 4 striatal targets	Completed
AAV	Aromatic L-amino acid decarboxylase (hAADC-2)	Parkinson’s disease	1/2	6 est/2fact	A Phase I/II Study of Intra-Putaminal Infusion of Adeno-Associated Virus Encoding Human Aromatic L-Amino Acid Decarboxylase in Subjects with Parkinson’s Disease	AADC Gene Therapy for Parkinson’s Disease	**NCT02418598** AAV-hAADC-2 is administered via bilateral intra-putaminal infusion. The number of vector genomes (vg) cohort 1: 3 × 10^11^ vg/subject cohort 2: 9 × 10^11^ vg/subject.	Terminated (Another clinical study for regulatory approval is planned)
AAV1	Neurotrophin factor 3 (NTF3)	Charcot–Marie–Tooth disease	1/2a	9est/0 fact	Phase I/II a Trial Evaluating scAAV1.tMCK.NTF3 for Treatment of Charcot–Marie–Tooth Neuropathy Type 1A (CMT1A)	Phase I/II a Trial of scAAV1.tMCK.NTF3 for Treatment of CMT1A	**NCT03520751** *N* = 3: intramuscular injection of (scAAV1.tMCK.NTF3) distributed bilaterally between both limbs at low dose (2 × 10^12^ vg/kg). *N* = 6: intramuscular injection of (scAAV1.tMCK.NTF3) distributed bilaterally between both limbs at low dose (6 × 10^12^ vg/kg)	Not yet recruiting
AAV2	Neurotrophic (growth) factor (Neurturin)	Parkinson’s disease	2	58 est/51 fact	Multicenter, Randomized, Double-Blind, Sham Surgery-Controlled Study of CERE-120 (Adeno-Associated Virus Serotype 2 [AAV2]-Neurturin [NTN]) to Assess the Efficacy and Safety of Bilateral Intraputaminal (IPu) Delivery in Subjects with Idiopathic Parkinson’s Disease	Double-Blind, Multicenter, Sham Surgery Controlled Study of CERE-120 in Subjects With Idiopathic Parkinson’s Disease	**NCT00400634**, CERE-120, bilaterally: 5.4 × 10^11^ vg	Completed
AAV2	Neurotrophic (growth) factor (Neurturin)	Parkinson’s disease	1	12 est	A Phase I, Open-Label Study of CERE-120 (Adeno-Associated Virus Serotype 2 [AAV2]-Neurturin [NTN] to Assess the Safety and Tolerability of Intrastriatal Delivery to Subjects with Idiopathic Parkinson’s Disease	Safety of CERE-120 (AAV2-NTN) in Subjects With Idiopathic Parkinson’s Disease	**NCT00252850**	Completed
AAV2	Human aromatic L-amino acid decarboxylase (AADC) gene	Parkinson’s disease	1	15 est/10 fact	An Open-label Safety and Efficacy Study of VY-AADC01 Administered by MRI-Guided Convective Infusion into the Putamen of Subjects with Parkinson’s Disease with Fluctuating Responses to Levodopa	Safety Study of AADC Gene Therapy (VY-AADC01) for Parkinson’s Disease (AADC)	**NCT01973543** VY-AADC01; Single dose, neurosurgically-infused, bilaterally into the striatum: 7.5 × 10^11^ vg, 1.5 × 10^12^ vg, 4.7 × 10^12^ vg	Active, not recruiting
AAV2	Human aromatic L-amino acid decarboxylase (AADC) gene	Parkinson’s disease	2	42 est	A Randomized, Placebo Surgery Controlled, Double-Blinded, Multi-center, Phase 2 Clinical Trial, Evaluating the Efficacy and Safety of VY-AADC02 in Advanced Parkinson’s Disease with Motor Fluctuations	VY-AADC02 for Parkinson’s Disease With Motor Fluctuations	**NCT03562494**, VY-AADC02 infusion, 2.5 × 10^12^	Recruiting
AAV2	Human aromatic L-amino acid decarboxylase (AADC) gene	Parkinson’s disease		50 est	An Observational, Long-Term Extension Study for Participants of Prior VY-AADC01 or VY-AADC02 Clinical Studies	Observational, Long-term, Extension Study for Participants of Prior VY-AADC01 or VY-AADC02 Studies	**NCT03733496**, Participants who received VY-AADC01 or VY-AADC02	Enrolling by invitation
AAV2	Human CLN2	Late infantile neuronal ceroid lipofuscinosis (LINCL)	1	11 est/10 fact	Administration of a Replication Deficient Adeno-Associated Virus Gene Transfer Vector Expressing the Human CLN2 cDNA to the Brain of Children with Late Infantile Neuronal Ceroid Lipofuscinosis	Safety Study of a Gene Transfer Vector for Children With Late Infantile Neuronal Ceroid Lipofuscinosis	**NCT00151216** AAV2CUhCLN2, *N* = 5, 3 × 10^12^ *N* = 6, 3 × 10^12^	Active, not recruiting
AAV2	Glial cell line-derived neurotrophic factor (GDNF)	Parkinson’s disease	1	28 est/25 fact	A Phase 1 Open-Label Dose Escalation Safety Study of Convection Enhanced Delivery (CED) of Adeno-Associated Virus Encoding Glial Cell Line-Derived Neurotrophic Factor (AAV2-GDNF) in Subjects with Advanced Parkinson’s Disease	AAV2-GDNF for Advanced Parkinson s Disease	**NCT01621581** 9 × 10^10^ vg, 3 × 10^11^ vg, 9 × 10^11^ vg 3 × 10^12^ vg	Active, not recruiting
AAV2	Human ND4	Leber’s congenital amaurosis	3	90 est	Efficacy and Safety of Bilateral Intravitreal Injection of GS010: A Randomized, Double-Masked, Placebo-Controlled Trial in Subjects Affected with G11778A ND4 Leber’s Hereditary Optic Neuropathy for Up to One Year	Safety and Efficacy Study of Gene Therapy for The Treatment of Leber’s Hereditary Optic Neuropathy	**NCT03293524**, GS010, IVT eye, 9 × 10^10^ vg	Active, not recruiting
AAV2	Human ND4	Leber’s congenital amaurosis	2 + 3	159 est/48 fact	Safety and Efficacy Study of Gene Therapy for The Treatment of Leber’s Hereditary Optic Neuropathy		**NCT03153293**, Single IVT injection, 1 × 10^10^ vg/0.05 mL	Active, not recruiting
AAV2	Human ND4	Leber’s hereditary optic neuropathy	3	36 est	A Randomized, Double-Masked, Sham-Controlled Clinical Trial to Evaluate the Efficacy of a Single Intravitreal Injection of GS010 in Subjects Affected for 6 Months or Less by LHON Due to the G11778A Mutation in the Mitochondrial ND4 Gene	Efficacy Study of GS010 for the Treatment of Vision Loss up to 6 Months From Onset in LHON Due to the ND4 Mutation (RESCUE)	**NCT02652767**, GS010	Active, not recruiting
AAV2	Human ND4	Leber’s hereditary optic neuropathy	3	37 est/36 fact	Randomized, Double-Masked, Sham-Controlled Clinical Trial to Evaluate the Efficacy of a Single Intravitreal Injection of GS010 in Subjects Affected for More Than 6 Months and to 12 Months by LHON Due to the G11778A Mutation in the ND4 Gene	Efficacy Study of GS010 for Treatment of Vision Loss From 7 Months to 1 Year From Onset in LHON Due to the ND4 Mutation (REVERSE) (REVERSE)	**NCT02652780**, rAAV2/2-ND4 intravitreal, 9 × 10^10^ vg	Completed
AAV2	Human ND4	Leber’s hereditary optic neuropathy		74 est	Long-Term Follow-Up of ND4 LHON Subjects Treated with GS010 Ocular Gene Therapy in the RESCUE or REVERSE Phase III Clinical Trials	RESCUE and REVERSE Long-term Follow-up (RESCUE/REVERSE)	**NCT03406104**, GS010, intravitreal injection	Recruiting
AAV2	Human ND4	Leber’s hereditary optic neuropathy			EAP Single Patient: Safety of Bilateral Intravitreal Injection of GS010 in a Single Subject Affected with G11778A ND4 Leber’s Hereditary Optic Neuropathy	EAP_GS010_single Patient	**NCT03672968**	Available
AAV2	Human ND4	Leber’s hereditary optic neuropathy	1	30 est/27 fact	An Open-Label Dose Escalation Study of an Adeno-Associated Virus Vector (scAAV2-P1ND4v2) for Gene Therapy of Leber’s Hereditary Optic Neuropathy (LHON) Caused by the G11778A Mutation in Mitochondrial DNA	Safety Study of an Adeno-associated Virus Vector for Gene Therapy of Leber’s Hereditary Optic Neuropathy (LHON)	**NCT02161380** scAAV2-P1ND4v2 intravitreal: 1.18 × 10^9^ vg 5.81 × 10^9^ vg 2.4 × 10^10^ vg 1.0 × 10^11^ vg	Recruiting
AAV9	CLN6	CLN6, Batten disease	1/2a	13 est/6 fact	Phase I/II a Gene Transfer Clinical Trial for Variant Late Infantile Neuronal Ceroid Lipofuscinosis, Delivering the CLN6 Gene by Self-Complementary AAV9	Gene Therapy for Children With Variant Late Infantile Neuronal Ceroid Lipofuscinosis 6 (vLINCL6) Disease	**NCT02725580** AT-GTX-501 (scAAV9.CB.CLN6)	Active, not recruiting
AAV9	CLN3	CLN3, neuronal ceroid- lipofuscinosis	1/2a	7 est	Phase I/II a Gene Transfer Clinical Trial for Juvenile Neuronal Ceroid Lipofuscinosis, Delivering the CLN3 Gene by Self-Complementary AAV9	Gene Therapy for Children With CLN3 Batten Disease	**NCT03770572** AT-GTX-502 High dose Low dose	Recruiting
AAV9	Human survival motor neuron (SMN)	Spinal muscular atrophy type 1 (SMA1)	1	15 est/9 fact	Phase I Gene Transfer Clinical Trial for Spinal Muscular Atrophy Type 1 Delivering AVXS-101	Gene Transfer Clinical Trial for Spinal Muscular Atrophy Type 1	**NCT02122952** iv *N* = 3, 6.7 × 10^13^ vg/kg *N* = 3, 2.0 × 10^14^ vg/kg	Completed
AAV9	Human survival motor neuron (SMN)	SMA	1	51 est/27 fact	Phase I, Open-Label, Dose Comparison Study of AVXS-101 for Sitting but Non-Ambulatory Patients with Spinal Muscular Atrophy	Study of Intrathecal Administration of Onasemnogene Abeparvovec-xioi for Spinal Muscular Atrophy (STRONG)	**NCT03381729** Intrathecal Onasemnogene abeparvovec-xioi 6.0 × 10^13^ vg 1.2 × 10^14^ vg 2.4 × 10^14^ vg	Suspended
AAV9	Human survival motor neuron (SMN)	Spinal muscular atrophy type 1 (SMA1)	3	22est/15 fact	Phase 3, Open-Label, Single-Arm, Single-Dose Gene Replacement Therapy Clinical Trial for Patients with Spinal Muscular Atrophy Type 1 With One or Two SMN2 Copies Delivering AVXS-101 by Intravenous Infusion	Gene Replacement Therapy Clinical Trial for Patients With Spinal Muscular Atrophy Type 1 (STR1VE)	**NCT03306277** iv Onasemnogene abeparvovec-xioi	Completed
AAV9	Human survival motor neuron (SMN)	Spinal muscular atrophy type 1 (SMA1)	3	44 est/30 fact	A Global Study of a Single, One-Time Dose of AVXS-101 Delivered to Infants with Genetically Diagnosed and Pre-Symptomatic Spinal Muscular Atrophy with Multiple Copies of SMN2	Pre-Symptomatic Study of Intravenous Onasemnogene Abeparvovec-xioi in Spinal Muscular Atrophy (SMA) for Patients With Multiple Copies of SMN2 (SPR1NT)	**NCT03505099** iv, Onasemnogene abeparvovec-xioi 1.1 × 10^14^ vg/kg	Active, not recruiting
AAV9	Human survival motor neuron (SMN)	Spinal muscular atrophy (SMA) type 1	3	33est/30 fact	European, Phase 3, Open-Label, Single-Arm, Single-Dose Gene Replacement Therapy Clinical Trial for Patients with Spinal Muscular Atrophy Type 1 with One or Two SMN2 Copies Delivering AVXS-101 by Intravenous Infusion	Single-Dose Gene Replacement Therapy Clinical Trial for Patients With Spinal Muscular Atrophy Type 1 (STRIVE-EU)	**NCT03461289** iv Onasemnogene Abeparvovec-xioi	Active, not recruiting
AAV10	Human SGSH and SUMF1 cDNAs	Sanfilippo type A syndrome	1 + 2	4 est	An Open-label, Single Arm, Monocentric, Phase I/II Clinical Study of Intracerebral Administration of Adeno-associated Viral Vector Serotype 10 Carrying the Human SGSH and SUMF1 cDNAs for the Treatment of Sanfilippo Type A Syndrome	Intracerebral Gene Therapy for Sanfilippo Type A Syndrome	**NCT01474343** Intracerebral SAF-301	Completed
AAV10	Human SGSH and SUMF1 cDNAs	Sanfilippo type A syndrome	1 + 2	4 est/	Long-Term Follow-Up of Patient with Sanfilippo Type A Syndrome Who Have Previously Been Treated in the P1-SAF-301 Clinical Study Evaluating the Tolerability and Safety of the Intracerebral Administration of SAF-301	Long-term Follow-up of Sanfilippo Type A Patients Treated by Intracerebral SAF-301 Gene Therapy	**NCT02053064** Intracerebral SAF-301 Long term effect	Completed
AAVrh10	Human apolipoprotein E2 (APOE2)	Alzheimer’s disease	1	15 est/0 fact	Maximum Tolerated Dose of Intracisternal delivery of AAVrh.10hAPOE2 (no results)	Gene Therapy for APOE4 Homozygote of Alzheimer’s Disease	**NCT03634007** AAVrh.10hAPOE2: 8.0 × 10^10^ gc/kg, 2.5 × 10^11^ gc/kg 8.0 × 10^11^ gc/kg	Recruiting
AAVrh10	Human CLN2	Late-infantile neuronal ceroid Lipofuscinosis	1	25 est/16fact	Direct CNS Administration of a Replication Deficient Adeno-associated Virus Gene Transfer Vector Serotype rh.10 Expressing the Human CLN2 cDNA to Children with Late Infantile Neuronal Ceroid Lipofuscinosis (LINCL)	Safety Study of a Gene Transfer Vector (Rh.10) for Children With Late Infantile Neuronal Ceroid Lipofuscinosis (LINCL)	**NCT01161576** AAVrh10.CUhCLN2, *N* = 6, 9 × 10^11^ *N* = 10, 2.85 × 10^11^	Active, not recruiting
AAVrh10	Human CLN2	Late infantile neuronal ceroid lipofuscinosis	1 + 2	16 est/8 fact	Improved Results on Weill Cornell LINCL Scale and Mullen Scale (no results posted)	AAVRh.10 Administered to Children With Late Infantile Neuronal Ceroid Lipofuscinosis	**NCT01414985** AAVrh10.CUhCLN2, *N* = 2, 9 × 10^11^ *N* = 6, 2.85 × 10^11^	Active, not recruiting

## Abbreviations

**AAV**	Adeno-associated virus
**Ad**	Adenovirus
**BBB**	Blood–brain barrier
**CNS**	Central neural system
**DNA**	Deoxyribonucleic acid
**GDNF**	Glial cell-line derived neurotrophic factor
**FGF**	Fibroblast growth factor
**HSPG**	Heparan sulfate proteoglycan
**HSV**	Herpes simplex virus
**NDD**	Neuro-degenerative disease
**PDGFR**	Platelet-derived growth factor receptor
**RNA**	Ribonucleic acid
**SFV**	Semliki forest virus
**SMN**	Survival motor neuron protein
**VEGF**	Vascular endothelial growth factor
